# CSVS, a crowdsourcing database of the Spanish population genetic variability

**DOI:** 10.1093/nar/gkaa794

**Published:** 2020-09-29

**Authors:** María Peña-Chilet, Gema Roldán, Javier Perez-Florido, Francisco M Ortuño, Rosario Carmona, Virginia Aquino, Daniel Lopez-Lopez, Carlos Loucera, Jose L Fernandez-Rueda, Asunción Gallego, Francisco García-Garcia, Anna González-Neira, Guillermo Pita, Rocío Núñez-Torres, Javier Santoyo-López, Carmen Ayuso, Pablo Minguez, Almudena Avila-Fernandez, Marta Corton, Miguel Ángel Moreno-Pelayo, Matías Morin, Alvaro Gallego-Martinez, Jose A Lopez-Escamez, Salud Borrego, Guillermo Antiñolo, Jorge Amigo, Josefa Salgado-Garrido, Sara Pasalodos-Sanchez, Beatriz Morte, The Spanish Exome Crowdsourcing Consortium, Fátima Al-Shahrour, Rafael Artuch, Javier Benitez, Luis Antonio Castaño, Ignacio del Castillo, Aitor Delmiro, Carmina Espinos, Roser González, Daniel Grinberg, Encarnación Guillén, Pablo Lapunzina, Esther Lopez, Ramón Martí, Montserrat Milá, José Mª Millán, Virginia Nunes, Francesc Palau, Belen Perez, Luis Pérez Jurado, Rosario Perona, Aurora Pujol, Feliciano Ramos, Antonia Ribes, Jordi Rosell, Eulalia Rovira, Jordi Surrallés, Isabel Tejada, Magdalena Ugarte, Ángel Carracedo, Ángel Alonso, Joaquín Dopazo

**Affiliations:** Clinical Bioinformatics Area, Fundación Progreso y Salud (FPS), Hospital Virgen del Rocío, Sevilla 41013, Spain; Bioinformatics in Rare Diseases (BiER), Center for Biomedical Network Research on Rare Diseases (CIBERER), ISCIII, Sevilla 41013, Spain; Computational Systems Medicine group, Institute of Biomedicine of Seville (IBIS) Hospital Virgen del Rocío, Sevilla 41013, Spain; Clinical Bioinformatics Area, Fundación Progreso y Salud (FPS), Hospital Virgen del Rocío, Sevilla 41013, Spain; Clinical Bioinformatics Area, Fundación Progreso y Salud (FPS), Hospital Virgen del Rocío, Sevilla 41013, Spain; Computational Systems Medicine group, Institute of Biomedicine of Seville (IBIS) Hospital Virgen del Rocío, Sevilla 41013, Spain; Functional Genomics Node, FPS/ELIXIR-ES, Hospital Virgen del Rocío, Sevilla 41013, Spain; Clinical Bioinformatics Area, Fundación Progreso y Salud (FPS), Hospital Virgen del Rocío, Sevilla 41013, Spain; Computational Systems Medicine group, Institute of Biomedicine of Seville (IBIS) Hospital Virgen del Rocío, Sevilla 41013, Spain; Functional Genomics Node, FPS/ELIXIR-ES, Hospital Virgen del Rocío, Sevilla 41013, Spain; Clinical Bioinformatics Area, Fundación Progreso y Salud (FPS), Hospital Virgen del Rocío, Sevilla 41013, Spain; Clinical Bioinformatics Area, Fundación Progreso y Salud (FPS), Hospital Virgen del Rocío, Sevilla 41013, Spain; Clinical Bioinformatics Area, Fundación Progreso y Salud (FPS), Hospital Virgen del Rocío, Sevilla 41013, Spain; Computational Systems Medicine group, Institute of Biomedicine of Seville (IBIS) Hospital Virgen del Rocío, Sevilla 41013, Spain; Clinical Bioinformatics Area, Fundación Progreso y Salud (FPS), Hospital Virgen del Rocío, Sevilla 41013, Spain; Computational Systems Medicine group, Institute of Biomedicine of Seville (IBIS) Hospital Virgen del Rocío, Sevilla 41013, Spain; Clinical Bioinformatics Area, Fundación Progreso y Salud (FPS), Hospital Virgen del Rocío, Sevilla 41013, Spain; Sistemas Genomicos, Paterna, Valencia 46980, Spain; Unidad de Bioinformática y Bioestadística, Centro de Investigación Príncipe Felipe (CIPF), Valencia 46012, Spain; Human Genotyping Unit–Centro Nacional de Genotipado (CEGEN), Human Cancer Genetics Programme, Spanish National Cancer Research Centre (CNIO), Madrid 28029, Spain; Human Genotyping Unit–Centro Nacional de Genotipado (CEGEN), Human Cancer Genetics Programme, Spanish National Cancer Research Centre (CNIO), Madrid 28029, Spain; Human Genotyping Unit–Centro Nacional de Genotipado (CEGEN), Human Cancer Genetics Programme, Spanish National Cancer Research Centre (CNIO), Madrid 28029, Spain; Edinburgh Genomics, The University of Edinburgh, Edinburgh EH9 3FL, UK; Department of Genetics, Instituto de Investigación Sanitaria-Fundación Jiménez Díaz University Hospital, Universidad Autónoma de Madrid (IIS-FJD, UAM), Madrid 28040, Spain; Department of Genetics, Instituto de Investigación Sanitaria-Fundación Jiménez Díaz University Hospital, Universidad Autónoma de Madrid (IIS-FJD, UAM), Madrid 28040, Spain; Center for Biomedical Network Research on Rare Diseases (CIBERER), ISCIII, Madrid 28040, Spain; Department of Genetics, Instituto de Investigación Sanitaria-Fundación Jiménez Díaz University Hospital, Universidad Autónoma de Madrid (IIS-FJD, UAM), Madrid 28040, Spain; Department of Genetics, Instituto de Investigación Sanitaria-Fundación Jiménez Díaz University Hospital, Universidad Autónoma de Madrid (IIS-FJD, UAM), Madrid 28040, Spain; Servicio de Genética, Ramón y Cajal Institute of Health Research (IRYCIS) and Biomedical Network Research Centre on Rare Diseases (CIBERER), Madrid 28034, Spain; Servicio de Genética, Ramón y Cajal Institute of Health Research (IRYCIS) and Biomedical Network Research Centre on Rare Diseases (CIBERER), Madrid 28034, Spain; Otology & Neurotology Group CTS 495, Department of Genomic Medicine, Centre for Genomics and Oncological Research (GENYO), Pfizer University of Granada, Granada 18016, Spain; Department of Otolaryngology, Instituto de Investigación Biosanitaria, IBS. GRANADA, Hospital Universitario Virgen de las Nieves, Universidad de Granada, Granada 18016, Spain; Otology & Neurotology Group CTS 495, Department of Genomic Medicine, Centre for Genomics and Oncological Research (GENYO), Pfizer University of Granada, Granada 18016, Spain; Department of Otolaryngology, Instituto de Investigación Biosanitaria, IBS. GRANADA, Hospital Universitario Virgen de las Nieves, Universidad de Granada, Granada 18016, Spain; Department of Maternofetal Medicine, Genetics and Reproduction, Institute of Biomedicine of Seville (IBIS), University Hospital Virgen del Rocío/CSIC/University of Seville, Seville 41013, Spain; Centre for Biomedical Network Research on Rare Diseases (CIBERER), Seville 41013, Spain; Department of Maternofetal Medicine, Genetics and Reproduction, Institute of Biomedicine of Seville (IBIS), University Hospital Virgen del Rocío/CSIC/University of Seville, Seville 41013, Spain; Centre for Biomedical Network Research on Rare Diseases (CIBERER), Seville 41013, Spain; Fundación Pública Galega de Medicina Xenómica, SERGAS, IDIS, Santiago de Compostela 15706, Spain; Navarrabiomed-IdiSNA, Complejo Hospitalario de Navarra, Universidad Pública de Navarra (UPNA), IdiSNA (Navarra Institute for Health Research), Pamplona, Navarra 31008, Spain; Navarrabiomed-IdiSNA, Complejo Hospitalario de Navarra, Universidad Pública de Navarra (UPNA), IdiSNA (Navarra Institute for Health Research), Pamplona, Navarra 31008, Spain; Undiagnosed Rare Diseases Programme (ENoD). Center for Biomedical Research on Rare Diseases (CIBERER), ISCIII, Madrid 28029, Spain; Fundación Pública Galega de Medicina Xenómica, SERGAS, IDIS, Santiago de Compostela 15706, Spain; Grupo de Medicina Xenómica, Centro de Investigación Biomédica en Red de Enfermedades Raras (CIBERER), CIMUS, Universidade de Santiago de Compostela, Santiago de Compostela, España; Navarrabiomed-IdiSNA, Complejo Hospitalario de Navarra, Universidad Pública de Navarra (UPNA), IdiSNA (Navarra Institute for Health Research), Pamplona, Navarra 31008, Spain; Clinical Bioinformatics Area, Fundación Progreso y Salud (FPS), Hospital Virgen del Rocío, Sevilla 41013, Spain; Bioinformatics in Rare Diseases (BiER), Center for Biomedical Network Research on Rare Diseases (CIBERER), ISCIII, Sevilla 41013, Spain; Computational Systems Medicine group, Institute of Biomedicine of Seville (IBIS) Hospital Virgen del Rocío, Sevilla 41013, Spain; Functional Genomics Node, FPS/ELIXIR-ES, Hospital Virgen del Rocío, Sevilla 41013, Spain

## Abstract

The knowledge of the genetic variability of the local population is of utmost importance in personalized medicine and has been revealed as a critical factor for the discovery of new disease variants. Here, we present the Collaborative Spanish Variability Server (CSVS), which currently contains more than 2000 genomes and exomes of unrelated Spanish individuals. This database has been generated in a collaborative crowdsourcing effort collecting sequencing data produced by local genomic projects and for other purposes. Sequences have been grouped by ICD10 upper categories. A web interface allows querying the database removing one or more ICD10 categories. In this way, aggregated counts of allele frequencies of the pseudo-control Spanish population can be obtained for diseases belonging to the category removed. Interestingly, in addition to pseudo-control studies, some population studies can be made, as, for example, prevalence of pharmacogenomic variants, etc. In addition, this genomic data has been used to define the first Spanish Genome Reference Panel (SGRP1.0) for imputation. This is the first local repository of variability entirely produced by a crowdsourcing effort and constitutes an example for future initiatives to characterize local variability worldwide. CSVS is also part of the GA4GH Beacon network.

CSVS can be accessed at: http://csvs.babelomics.org/.

## INTRODUCTION

Sequencing technologies have experienced an unprecedented development during the last decade (1) that resulted in different international collaborative projects (2–4) which contributed to an extraordinary increase in the knowledge of the mutational spectrum of diseases. This generation of knowledge has been especially significant in diseases with high morbidity and mortality, caused by highly penetrant (typically protein-coding) variants (5,6). In fact, more than 4500 monogenic diseases can nowadays be directly diagnosed by personalized genomics (7), a possibility that might soon be extended to the whole spectrum of rare diseases with a genetic background (8). Among the strategies used to discover new disease variants, especially in monogenic disorders, frequency-based filtering has demonstrated to be a very useful tool (9). The rationale is as follows: variants that are relatively common in a control population (common variation) are likely benign (10), while rare variants (especially if they have functional consequences) found in multiple affected cases but absent in the control population are likely to cause disease (11–13). These filters search for genes or variants present in all (or most) affected individuals but in none (or very few) of the unaffected control individuals. Therefore, it seems clear that the availability of healthy controls is a decisive factor for the progress of discovery of new disease determinants.

From an historical perspective, the 1000 Genomes Project produced the first comprehensive catalogue of common human genetic variation (14). However, it is known that low frequency (with minor allele frequencies, MAF, under 5%) and rare (MAF under 0.5%) variants, typically population-specific (15), are poorly represented in such catalogue (14). Actually, recent studies have described a remarkable local component (16–18) and a high stratification level (19,20) in many rare variants with uncertain functional consequences. As a consequence of this, the risk of many diseases differs in distinct human populations according to their genetic backgrounds (21,22). In fact, the knowledge of the genetic variability of the local population has been revealed as a critical factor for the discovery of new disease variants (23). All these observations highlight the need for population-specific catalogues of genetic variation (24). However, only a few initiatives to study genetic variation at the population level have been carried out to date, which include a whole-genome sequence (WGS) study of 100 Malays (25), the *Genome of the Netherlands*, with low-resolution (∼13×) WGS data of 250 trio-families from across the entire country (15), the French-Canadians study of 109 exomes (26), the Medical Genome Project that produced a catalog of the healthy Spanish population with almost 270 exomes (23), the 3000 Finnish genomes (27) and the Icelandic population study of medium resolution (∼20×) WGS of 2636 individuals (28) or the high resolution (>30×) WGS of 1070 healthy Japanese individuals (29) and the recent genetic analysis of the Iranian population (30).

In spite of its recognized usefulness, large-scale sequencing projects of cohorts of local ‘healthy’ populations require expensive consortium-based projects to obtain a representative sample of the population targeted. Unfortunately, funding bodies that are prone to support research on diseases, tend to be, however, reluctant to fund projects that involve systematic sequencing of healthy individuals. In this scenario, a crowdsourcing strategy can provide a feasible alternative to traditional working schemas by organizing consortia that collect data from different groups that ultimately are collectively benefited of the sample size cooperatively obtained. Crowdsourcing is becoming a very popular strategy in biomedicine (31) and can be defined as ‘the process of getting services, information, labor or ideas by outsourcing through an open call, especially through the Internet’ (32). Recently some examples of crowdsourced research have demonstrated an increased accuracy in predicting breast cancer survival (33), response to drugs (34) or to toxic compounds (35) from both, clinical and genomic data, and show how ‘crowdsourced data science challenges can achieve in months what would take years through conventional research approaches’ (36).

## MATERIALS AND METHODS

### Subjects

The database contains detailed allelic frequencies corresponding to The MGP population, sequenced in the context of the Medical Genome Project (http://www.clinbioinfosspa.es/content/medical-genome-project), which includes 267 healthy, unrelated samples of Spanish origin (EGA, accession: EGAS00001000938), other healthy controls, patients of different diseases, accompanied in some cases of unrelated phenotypically healthy carriers. The sequences were contributed by different consortiums and projects, including groups from the Spanish Network for Research in Rare Diseases, CIBERER, results from the EnoD, (Undiagnosed Rare Diseases programme; https://www.ciberer.es/en/transversal-programmes/scientific-projects/undiagnosed-rare-diseases-programme-enod), the Project Genome 1000 Navarra (NAGEN 1000; (https://www.nagen1000navarra.es/en), The RareGenomics (https://www.rare-genomics.com/) from Madrid, and other research groups and initiatives across Spain (37,38), which currently sum up a total of 2027 genomic and exomic sequences of unrelated Spanish individuals.

### Testing sample locality

Ensuring the Spanish locality of the samples uploaded in the CSVS is key for the project. Here, we specifically developed a methodology to double-check the origin of each sample. Sequences belonging to different populations in the 1000 genomes project (14) were used to train a Machine Learning based decision model to discriminate Spanish samples from the rest of populations. Firstly, SNPs corresponding to the genomic regions shared by all the samples having a MAF > 0.01 were selected. Then, individual ancestry in 1000 genomes was estimated for 26 subpopulations using ADMIXTURE (39). Therefore, each individual is described by a vector of 26 features that correspond to the probabilities of belonging to any of the 26 subpopulations of 1000 genomes. Then, a machine learning binary classificatory was built using a well-known variant of the gradient boosting machine: extreme gradient boosting (*XGBoost*) (40) (see Supplementary Methods for details).

### Testing sample kinship and outlier sample detection

A test to determine undesired samples based on their percentage of novel variants introduced in the database, either by excess (potential noisy sample) or by defect (close relative or individual already in the database), has also been used to populate the CSVS database. A leave-one-out cross-validation (LOOCV) strategy was to build a distribution of percentages of variants contributed by any single sample to the pool of variants present in the rest of the database. Samples were considered potential outliers if overpass 1.5 times the interquartile range from first and third quartile in the distribution obtained (see Supplementary Methods for details).

### Construction of the reference imputation panel

Two alternative reference panels were created for comparison purposes that include the CSVS WGS variant panel composed of 228 samples plus: (i) the entire 1000G reference panel (CSVS+1000G) and (ii) exclusively the Spanish population (IBS subpopulation) contained in the 1000G panel (CSVS+IBS), using the *Minimac3* imputation tool (41). The four longest chromosomes (chromosome 1–4) were used to estimate the correlation between real and imputed genotypes (*r*^2^ parameter) and assess the imputation accuracy (see Supplementary Methods for details).

## RESULTS

### The CSVS database

**Figure 1.**
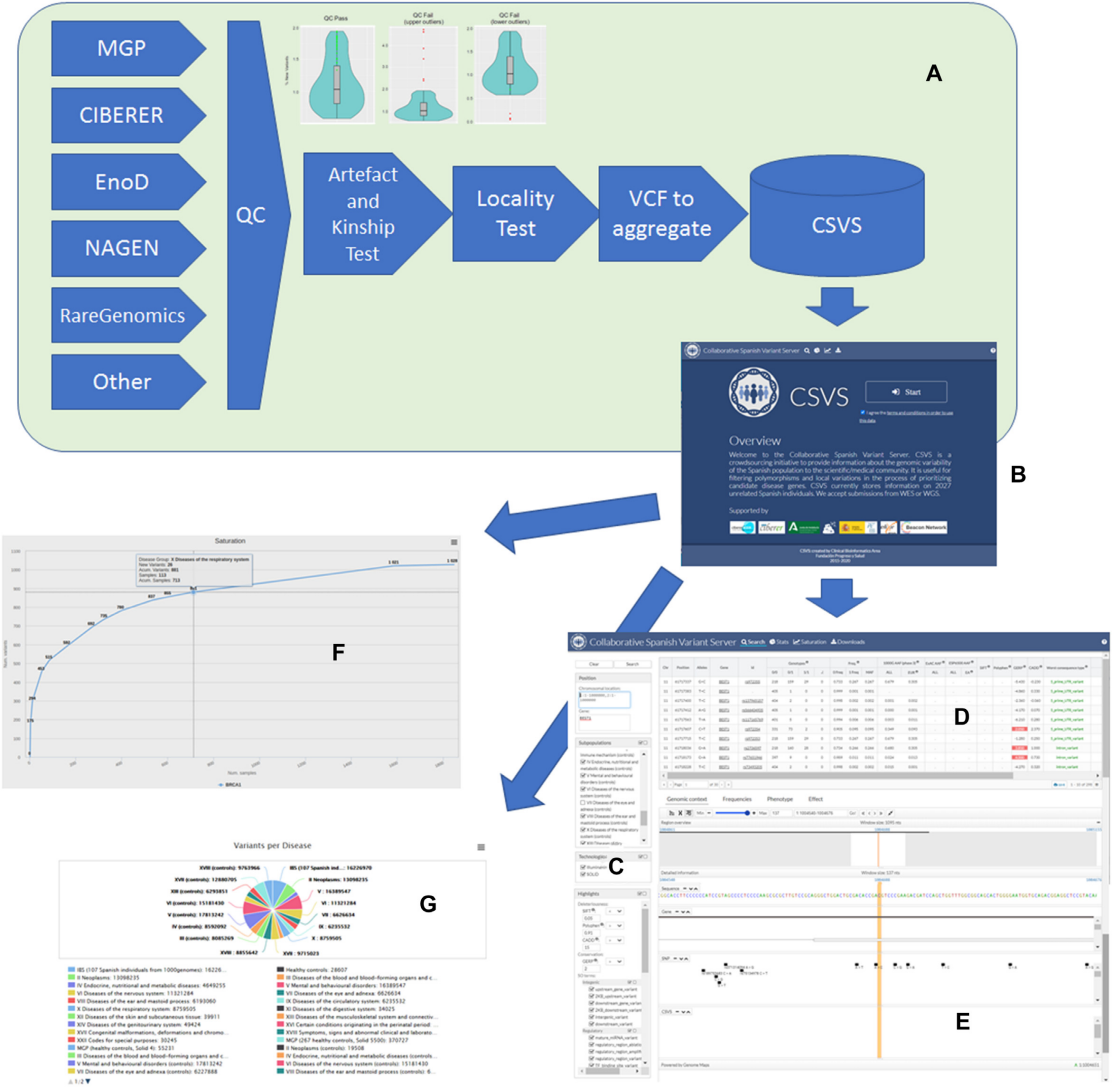
(**A**) data is contributed by different genomic projects and pass through different quality control steps including an artefact and kinship test (that detects upper outliers, with an unexpected high ratio of private variants, most likely errors, and lower outliers, that are duplicates or close kinship individuals) and locality test before being inserted in the database. (**B**) Initial CSVS page. (**C**) Query panel in the Search option. (**D**) List of variants found in the Spanish population within the selected region along with complementary information on impact, conservation, other's population frequencies and phenotype. (**E**) genomic browser that displays the selected variant in its genomic context. (**F**) Saturation plot. (**G**) Updated contents of the database.

Figure 1A shows how data contributed by different genomic projects undergo different quality control steps, including an artifact and kinship detection tests and locality test, described above. Then the original VCFs are aggregated as counts of variants, binned by ICD10 (https://www.icd10data.com/) disease categories, and inserted in the CSVS database.

### The CSVS interface

The initial screen (Figure 1B) requires the acceptance of the ‘Terms and conditions for the use of the CSVS database’ (http://csvs.babelomics.org/downloads/CSVSTermsAndConditions_use.pdf) before starting any operation. Once accepted, different options can be used.

#### The search option

This is the main option and allows querying the CSVS database. In the left panel (Figure 1C) queries can be done by gene symbol or by chromosomal regions. Also, one or several disease categories can be excluded, and variants can be highlighted using different types of scores (e.g. SIFT (42), Polyphen (43), CADD (44), Gerp (45)) as well as Sequence Ontology terms for the variation consequences.

The results of the query (Figure 1D) include a list of the positions for which variation has been found in the Spanish population along with complementary data as: chromosome, position, reference allele and alternative allele, allelic frequencies in the Spanish population, allelic frequencies in the 1000 genomes populations and in the EVS populations, impact and conservation indexes (SIFT, Polyphen, CADD, Gerp), the wort of the consequence types assigned to the mutation and the phenotypes, corresponding to known clinical information for the variants, extracted from ClinVar (46), COSMIC (47) and are annotated interactively on each query using the CellBase (48) webservices. Also a visualization of the variant in the genomic context is provided, based on the Genome Maps browser (49). Additionally, some extra detailed information can be found on the population frequencies observed for the variant, the phenotype or the effect.

#### Contact request

An interesting option is the *Contact request* button, offered for any variant in the query results panel, which is a local equivalent of a Matchmaker exchange service (50), extensively used to contact the original contributor of a specific sequence.

#### Saturation plots

**Figure 2.**
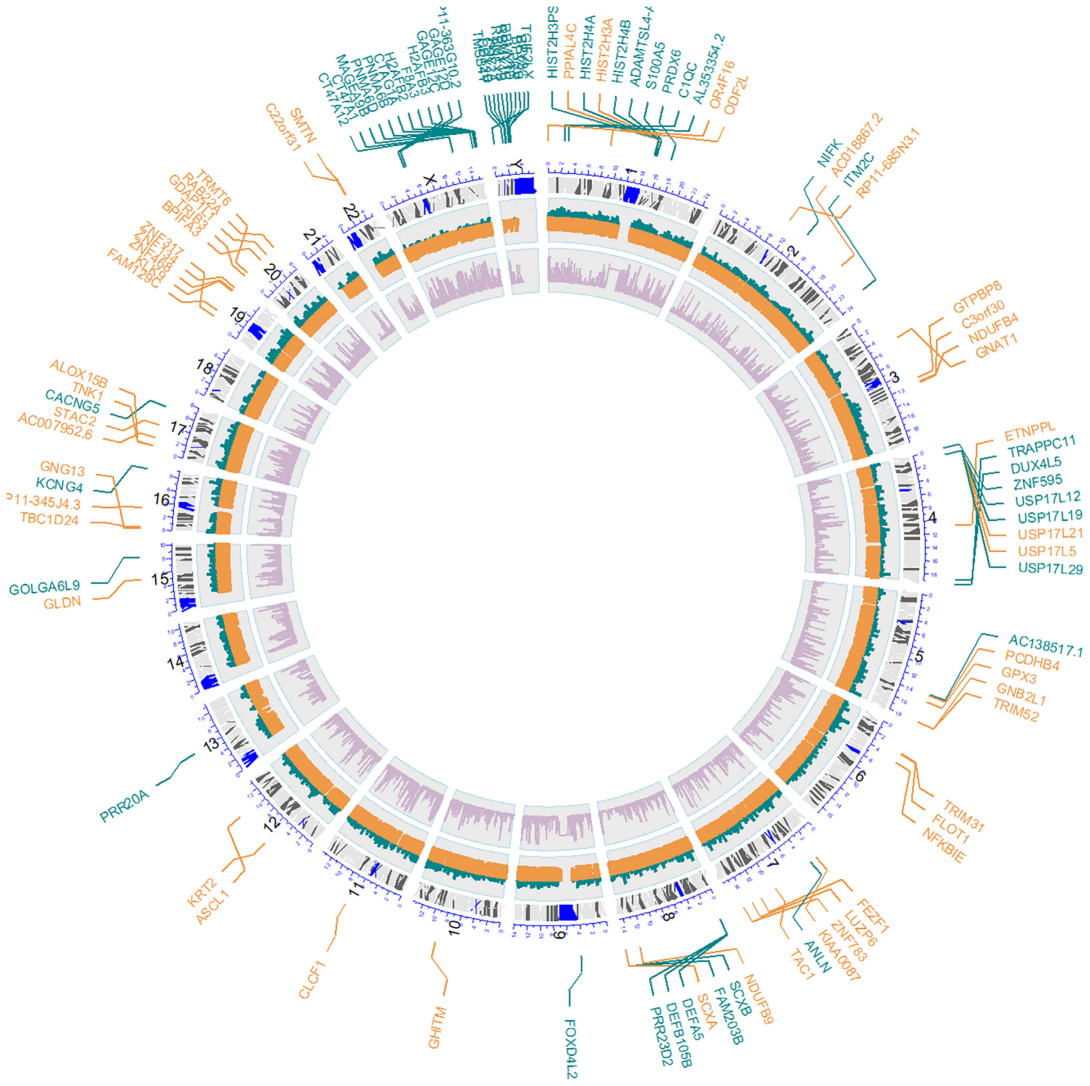
Circos plot showing the different genes with high saturation (orange) and low saturation (green) along the chromosomes, which were significantly enriched in functional terms in Supplementary Figure S1.

Saturation plots (Figure 1F) provide an interesting perspective on the general conservation of the gene studied and, consequently on the possibilities of discovering new variants into it. Genes highly constrained to change will saturate soon and a relatively low number of individuals will capture most of the tolerated mutation the gene can handle, while unconstrained genes will present a still growing slope, meaning that there are still many variants that can potentially be discovered. Discovering a new variant in a saturated gene (constrained to change) can be more relevant than the same finding in a non-saturated gene (unconstrained). Saturation has a clear functional component, that can easily be revealed by enrichment analysis of the genes ranked by saturation. Thus, when genes are ranked by their relative saturation, enrichment analysis using **enrichR** (51) shows how highly saturated genes (constrained) are enriched in functional terms related to meiosis, cell signaling, proliferation and homeostasis, while the less saturated (unconstrained) are more related to sensory perception, immune response and similar functionalities (see Supplementary Results and Supplementary Figure S1). Figure 2 depicts how genes with high and low saturation are distributed along the chromosomes. Interestingly, sex chromosomes seem to be enriched in low saturated genes.

#### Downloads and statistics

Partial or total downloads of the aggregated data are possible upon the reception of the corresponding data download agreement duly signed.

The **Stats** option provides an updated view of the content of the CSVS database.

### The Spanish Genome Reference Panel (SGRP1.0)

Supplementary Figure S2 shows the accuracy of the two reference panels derived for imputation in the Spanish population. Both reference panels including the CSVS WGS reference outperformed the 1000 genomes reference. The imputation accuracy increases when variants in rare sites were included (MAF > 0.005). The most realistic imputation panel includes CSVS and the IBS population of the 1000 genomes.

### Variants of pharmacogenomic interest

Interindividual genetic variability in genes involved in drug-metabolizing enzymes and transporters have been linked to differences in the efficacy and toxicity of many medications: Moreover, genetic differences between human populations are becoming increasingly recognized as important factors accounting for interindividual variations in drug responsiveness (52,53). Approximately one-fifth of new drugs approved in the past years demonstrated differences in response across ethnic groups, leading to population-specific prescribing recommendations (54). In spite of the consensus about the existence of a relative homogeneity within European populations, population-specific differences in the Spanish population were recently reported (23). Using the individuals of the CSVS repository, we addressed how population-specific differences in those genes involved in drug Absorption, Distribution, Metabolism, Excretion and Toxicity (ADMET) could affect in the rates and risks for drug inefficacy and/or adverse drug reactions in the Spanish population. We estimated the allele frequencies of a total of 142 pharmacogenetic variants described in the PharmGKB database (55) with pharmacogenetic clinical recommendations (PharmGKB variants level 1A and 1B) and a total of 40 of these were found to be polymorphic in the CSVS. When compared with the allele frequencies calculated from genetic data of 30 000 European non-Finnish individuals (gnomAD (56)), no relevant frequency differences between the general European population and the Spanish population were observed, being the most different rs2228001 (level 1B) in XPC gene, rs2108622 (level 1A) in CYP4F2 gene and rs3892097 (level 1A) In CYP2D6 gene (*P*-value ≤ 1 × 10^−10^). Regarding the non-polymorphic variants, we observed that all of them are low-frequency variants (lower than 0.00065) and we do not expect to find a heterozygous individual due to the sample size in our repository (Supplementary Table S1).

Apart from the genetic variants already recommended to be implemented in the clinical setting, it was found that genetic variability with functional impact was governed by few high-frequency variants for some genes, but the functionality of the majority of pharmacogenes is dominated by rare genetic variants (57). In addition, local variability in these ADMET genes could also be very relevant for explaining a substantial part of the unexplained inter-individual differences in drug response and toxicities at the population-specific level, so that it is mandatory to have available population-specific catalogs of these pharma-variants (mainly rare) to explore their contribution to predictions of drug response. To examine this, we studied the variability of the Spanish population captured by our repository in a total of 421 well-known pharmacogenes involved in drug pharmacokinetics and/or drug response (Supplementary Table S2). High-impact variants within those pharmacogenes were defined according to the Variant Effect Predictor (58) as those having having the following consequence types: frameshift, splice acceptor, splice donor, start lost, stop gained, stop lost, transcript ablation and transcript amplification. Additionally, deleterious missense variants categorized as deleterious by CONDEL (59) or having a LoFtool score (60) lower than the first quartile corresponding to the most intolerant variants.

As before, the same comparison with the corresponding European non-Finnish variants rendered a total of 318 high impact variants and 235 likely deleterious missense single nucleotide variants in the pharmacogenes studied. Interestingly, 18 (5.6%) high impact variants and 18 (7.6%) missense variants identified were present in our Spanish population while no heterozygotes were observed in these positions across ∼30 000 healthy individuals of the European non-Finnish population. Also, a non-negligible percentage of private variation was observed in these genes encoding proteins involved in drug metabolism, transport, and response, and this information can be used to pinpoint relevant private genetic variants to be included in the design of population-specific pharmacogenetic genotyping arrays to be utilized in the implementation of pharmacogenetic diagnostics in the clinical setting (Supplementary Table S3).

### CSVS Beacon

Since 2017, CSVS makes its genomic information discoverable through the GA4GH Beacon network (https://beacon-network.org/). In order to improve the performance of the CSVS Beacon API we set up an SQLite database specific for this purpose. Although CSVS stores data in 1-base it can respond to queries in both 1-base or 0-base (Beacon requests data in 0-base). A form to directly make Bacon-style queries is also available (http://ucscbeacon.clinbioinfosspa.es/).

## DISCUSSION

The genetic variability of the local population is recognized as one of the most relevant factors in the discovery of new disease variants, especially in mendelian diseases (6,8,23). However, genomic data of healthy individuals belonging to the local population of interest are often scarce when not unavailable. The CSVS provides an original solution to this problem. The CSVS is a continuously growing resource that collects genomic or exomic sequences of the Spanish local population, no matter whether these come from healthy or diseased individuals. The main objective is using the repository as a pseudo-control population for finding new disease-causing variants and genes, with the idea that ‘disease A is a healthy control for disease B’. Despite gene pleiotropy cannot be completely ruled out, data are binned at higher disease ICD10 categories, where this gene property can be considered negligible. Actually, resources like Disgenet (61) can be used in case of doubt, and will be incorporated to automatically exclude the proper disease categories, in future CSVS versions. Since the collection of population-specific genomic data from individuals with different diseases are easier to collect than those from healthy donors, CSVS provides an example for the construction of population-specific pseudo-control repositories by means of crowdsourcing (31). Moreover, the CSVS Beacon and the *Contact request* option makes of CSVS a tool with high potential of discoverability. Thus, CSVS sets the ground and it is an example for future federated European infrastructures (62).

## DATA AVAILABILITY

CSVS is an open resource available at http://csvs.babelomics.org/.

The CSVS code, as well as the code of the different tests used is available in the corresponding github repository: https://github.com/babelomics/CSVS.

## Supplementary Material

gkaa794_Supplemental_Files
