# Supplementary material for: CSVS, a crowdsourcing database of the Spanish population genetic variability
Source: Nucleic Acids Res. 2020 Sep 29;49(D1):D1130–7. doi: 10.1093/nar/gkaa794 (PMC7778906; doi:10.1093/nar/gkaa794)
Supplement: gkaa794_Supplemental_Files [file gkaa794_supplemental_files.zip › CSVS Supplementary.R1.pdf]

CSVS, a crowdsourcing database of the Spanish population genetic variability

María Peña-Chilet, Gema Roldán, Javier Perez-Florido, Francisco M. Ortuño, Rosario Carmona, Virginia Aquino, Daniel Lopez-Lopez, Carlos Loucera, Jose L. Fernandez-Rueda, Asunción Gallego, Francisco García-Garcia, Anna González-Neira, Guillermo Pita, Rocío Núñez-Torres, Javier Santoyo-López, Carmen Ayuso, Pablo Minguez, Almudena Avila-Fernandez, Marta Corton, Miguel Ángel Moreno-Pelayo, Matias Morin, Alvaro Gallego-Martinez, Jose A. Lopez-Escamez, Salud Borrego, Guillermo Antiñolo, Jorge Amigo, Josefa Salgado-Garrido, Sara Pasalodos-Sanchez, Beatriz Morte, The Spanish Exome Crowdsourcing Consortium, Ángel Carracedo, Ángel Alonso, Joaquín Dopazo

Contents

Supplementary Materials 2
- Testing locality 2
- Kinship and novel variant test 3
- Construction of the Spanish Reference Imputation Panel 4

Supplementary Results 5
- Saturation plots 5
  - Supplementary Figure 1 5
- Imputation accuracy 6
  - Supplementary Figure 2 6

Supplementary References 7

Supplementary Materials

Testing locality

Ensuring the Spanish locality of the samples uploaded in the CSVS is key for the project. Here, we specifically developed a methodology to double-check the origin of each sample. Sequences belonging to different populations in the 1000 genomes project (1) were used to train a Machine Learning based decision model to discriminate Spanish samples from the rest of populations.

Since CSVS is composed of WGS as well as WES and clinical exomes, capturing different regions of the genome, only SNPs located on the maximum common captured region (MCCR) across all the set of samples were initially used. BCFtools (2) and custom bash scripts were used to build the MCCR. Although restricting the biomarkers used to only SNPs within the MCCR could decrease the prediction accuracy in some samples (especially in the case of WGS), the use of a common training set provides a unified framework for the comparison between samples and the possibility to choose a unique reference threshold. Rare SNPs (MAF<0.01) were also removed to reduce the number of biomarkers used to train the model and, consequently, the computation time. Then, individual ancestry in 1000 genomes phase 3 samples (reference panel) for 26 subpopulations was estimated using ADMIXTURE (3). The goal was to learn population structure from the 1000 genomes reference panel and project the CSVS individuals on it. In spite of the fact that the projection over a population is done on a sample-basis (the inferred structure for each sample is independent on the rest of the population itself), ADMIXTURE expects variability (at least one sample with non-reference allele) for each biomarker used to train the model. To overcome this limitation, projections over a virtual test population containing the test sample plus a synthetic sample containing non-reference alleles for each position were made.

Any sample is described by a vector with 26 features that correspond to the probabilities of belonging to any of the 26 subpopulations of 1000 genomes computed with ADMIXTURE. In addition, as a result of the methodology used, the sample space is divided into two clearly differentiated sets, namely, training (Tr) and test (Ts), which correspond to the initial stratification and projections, respectively. In summary, the ML method computes a probability for each sample, where the closer to 1, the greater the likelihood the sample will be of Spanish origin.

First, a binary classifier is constructed using a well-known variant of the gradient boosting machine: extreme gradient boosting (XGBoost)(4). The model takes as input the probabilities computed during the population stratification, where the positive class is made up of those samples labeled "Iberian", while everything else is labeled negative. As previously written, the

training is done with the subset Tr. As previously shown (5), the hyper-parameters of a particular ensemble are specific to each problem and domain, so its optimization is still an open problem. In our case, in order to find a "quasi-optimal" set of hyper-parameters for XGBoost, we use a sequential stochastic optimization algorithm known as Tree-structured Parzen Estimator (TPE) (6), where the main XGBoost hyperparameters form the set of solutions, while the function to be optimized is the result of averaging the area under the Precision-Recall curve (AUCPR) when performing K-fold cross-validation on Tr (with k = 5). The machine learning procedure has been implemented in Python(3.7), on top of the *scikit-learn* (7) and the *hyperopt* (8) libraries. The code used for this test can be found here: https://github.com/babelomics/CSVS.

Kinship and novel variant test

A test to determine undesired samples based on their percentage of novel variants introduced in the database, either by excess (noisy sample) or by defect (close relative or individual already in the database), has also been used to populate the CSVS database. A variant is tagged as novel if that variant has not been identified before in any of the remaining samples in the database. The proposed model underlying this test is designed by measuring the percentage of novel variants which each sample has contributed to the database in order to establish the expected distribution. This model has been developed with a two-fold objective:

i) to determine and filter out samples with a specific kinship with other samples already included in the database as well as duplicities (more than one sample coming from the same individual). These samples are expected to produce significantly low percentages of novel variants (lower outlier) as found variants will be commonly shared with the already introduced relatives.

ii) to identify and avoid potentially erroneous samples producing exceedingly high percentages of novel variants (upper outlier) and, therefore, introducing biased data into the database. These outliers can be produced by an excessively noisy sample, due to errors in the sequencing or analysis steps or by specific characteristics not matching with the purpose of the database (origin, ethnicity, etc.) In that last case, the outlier will be confirmed by the locality test previously described.

In order to prepare and validate the model, a leave-one-out cross-validation (LOOCV) strategy was performed. For each test sample, a database of variants was built with the remaining samples and the percentage of novel variants from that test sample was obtained. This percentage is measured as the number of novel variants divided by the total number of variants of that specific sample. The percentages calculated for every sample in the database are then applied to generate the normal distribution and to identify potential outliers. Similar to boxplot

graphs, one value is considered outlier if overpassing 1.5 times the interquartile range (IQR) from first and third quartile in the distribution, respectively. When a new dataset is going to be included in the database, samples are first tested with the current model. Just after, the model is re-trained and a new normal distribution is calculated including the samples passing this test.

Since the database collects both WGS and WES samples, a particular model has been performed for each of these sample types. Specifically, given WES samples come from different capture kits, a maximum common capture regions (MCCR) has been developed to identify common regions among all the kits, similarly to the locality test. These models have been developed in Python (3.7) using the **scikit-allele** library[1] to retrieve variants from VCF format. The outlier detection based in the models is written in R language and can be found in the github repository: https://github.com/babelomics/CSVS.

Construction of the Spanish Reference Imputation Panel

From the CSVS cohort, 228 whole-genome sequencing samples were considered to create an accurate Spanish reference imputation panel. SNPs and INDELs were selected for this reference panel by including only non-singleton variants (AC≥2) and adding them to the 1000G Phase 3 panel provided by the *Minimac3* imputation tool (9). Two alternative reference panels were proposed for comparison purposes depending on the added 1000G subpanel: i) the CSVS WGS variant panel adding the entire 1000G reference panel (CSVS+1000G); and ii) exclusively the Spanish population (IBS subpopulation) contained in the 1000G panel (CSVS+IBS). The Spanish WGS cohort from CSVS was first pre-phased by using the *SHAPEIT* software (10). Subsequently, both Spanish reference panels were generated by the *Minimac3* imputation software (9). These panels already included the parameter estimation step for speeding up the imputation process (reference panels in M3VCF format).

Both generated imputation panels were then compared against the original 1000G Phase3 panel. For testing purposes, 196 WGS samples from CSVS were considered for creating the reference panels whereas the remaining 32 WGS samples were kept for testing the imputation process. Variant calling (including joint genotyping steps) was separately performed in both WGS subsets (reference and testing) to assure their independence. The imputation test was performed on the four longest chromosomes (chromosome 1-4). Variants (both SNPs and INDELs) were gathered by their MAF. The estimated correlation between real and imputed genotypes (r^2 parameter) was applied for the imputation accuracy assessment.

---

[1] https://github.com/cggh/scikit-allel

Supplementary Results

Saturation plots

Saturation plots provide a visual representation of the number of new mutations contributed by increasing numbers of sequences. Genes highly constrained to change will saturate soon and a relatively low number of individuals will capture most of the tolerated mutation the gene can handle while unconstrained genes will present a still growing slope, meaning that there are still many variants that can potentially be discovered. Obviously finding new variants in constrained genes will be more relevant than in unconstrained ones.

Interestingly, when genes are ranked by their relative saturation, enrichment analysis using **enrichR** (11) shows that saturated genes (constrained) are enriched in functional terms related to meiosis, cell signaling, proliferation and homeostasis, while the less saturated (unconstrained) are more related to sensory perception, immune response and similar functionalities (See Supplementary Figure 1)

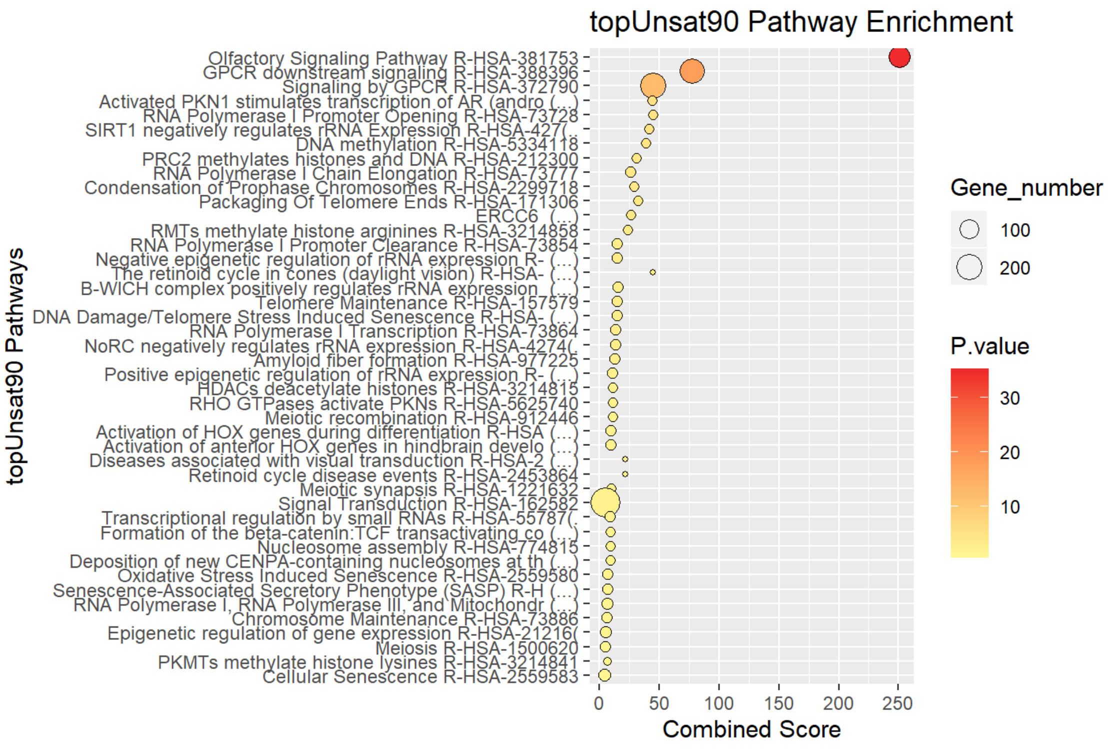

Supplementary Figure 1: Enrichment analysis with **enrichR** using Reactome functional terms.

Imputation accuracy

A subset test of 32 WGS samples from Spanish population were imputed with the two generated reference panels. As shown in Supplementary Figure 2, both reference panels including the CSVS WGS reference outperformed the 1000 genomes reference in terms of genotype correlation (r^2) for chromosomes 1-4, independently on the MAF in the tested variants.

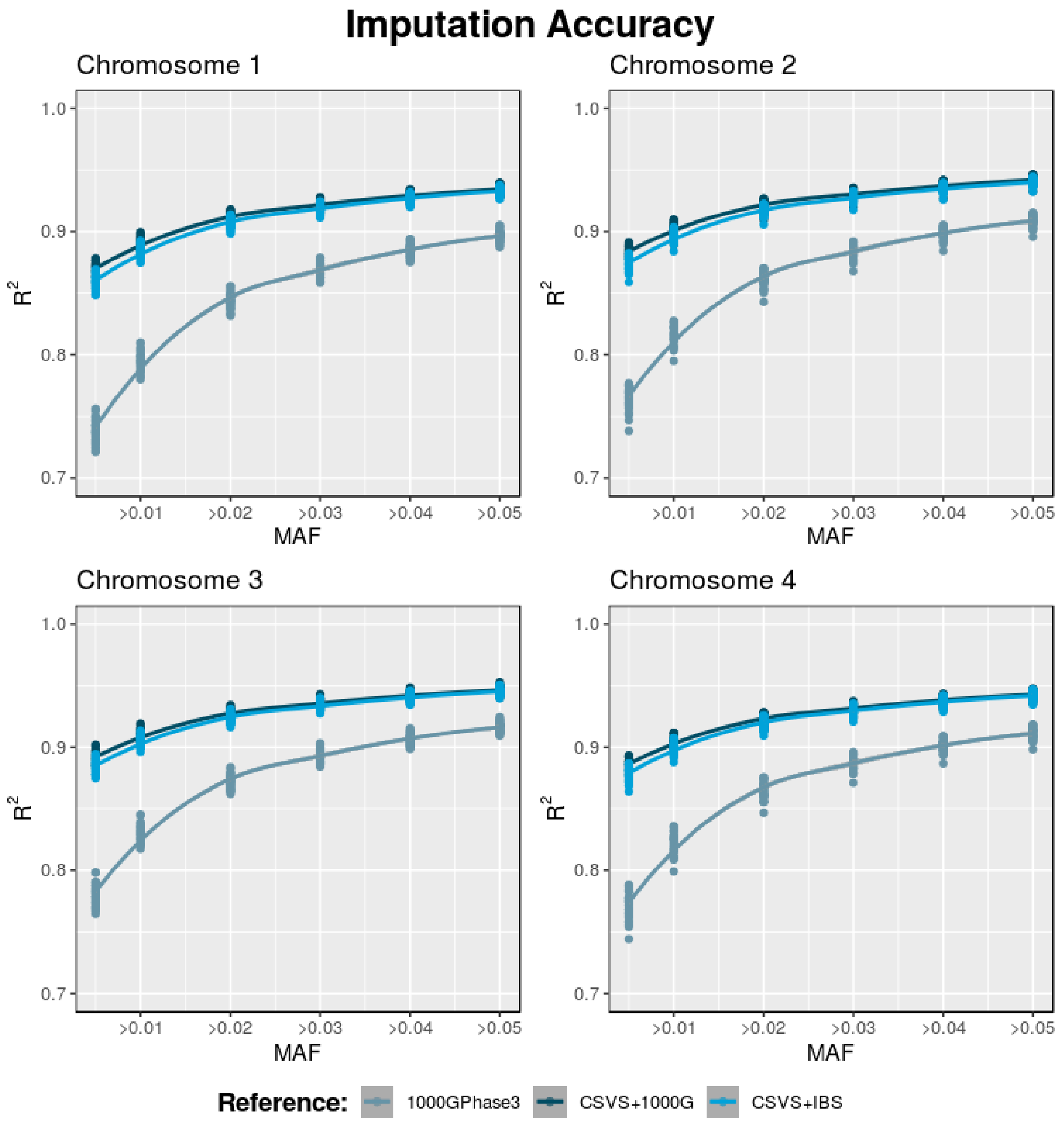

Supplementary Figure 2. Genotype correlation (r^2) for chromosomes 1-4 for SNPs / indels at different MAFs in the three reference panels built, based on 1000 genomes, CSVS + 1000 genomes and CSVS + IBS population from 1000 genomes.

The imputation improvement is even more significant when variants in rare sites were included (MAF > 0.005), increasing the accuracy from 75%-80% to almost 90% in the four chromosomes. Also, there were no significant differences in terms of imputation accuracy between both

Spanish references (adding only IBS subpopulation or the entire 1000G reference panel to our CSVS population). However, including only IBS makes this imputation panel a more realistic Spanish reference since it is exclusively generated from Spanish datasets and, therefore, it better represents the variants and polymorphisms of this population.

Supplementary References

1. Abecasis, G.R., Auton, A., Brooks, L.D., DePristo, M.A., Durbin, R.M., Handsaker, R.E., Kang, H.M., Marth, G.T. and McVean, G.A. (2012) An integrated map of genetic variation from 1,092 human genomes. *Nature*, **491**, 56-65.
2. Li, H., Handsaker, B., Wysoker, A., Fennell, T., Ruan, J., Homer, N., Marth, G., Abecasis, G. and Durbin, R. (2009) The Sequence Alignment/Map format and SAMtools. *Bioinformatics*, **25**, 2078-2079.
3. Alexander, D.H., Novembre, J. and Lange, K. (2009) Fast model-based estimation of ancestry in unrelated individuals. *Genome research*, **19**, 1655-1664.
4. Chen, T. and Guestrin, C. (2016), *Proceedings of the 22nd acm sigkdd international conference on knowledge discovery and data mining*. ACM, pp. 785-794.
5. Wolpert, D.H. and Macready, W.G. (1997) No free lunch theorems for optimization. *IEEE transactions on evolutionary computation*, **1**, 67-82.
6. Bergstra, J.S., Bardenet, R., Bengio, Y. and Kégl, B. (2011), *Advances in neural information processing systems*, pp. 2546-2554.
7. Pedregosa, F., Varoquaux, G., Gramfort, A., Michel, V., Thirion, B., Grisel, O., Blondel, M., Prettenhofer, P., Weiss, R. and Dubourg, V. (2011) Scikit-learn: Machine learning in Python. *Journal of machine learning research*, **12**, 2825-2830.
8. Bergstra, J., Yamins, D. and Cox, D. (2013), *International Conference on Machine Learning*, Vol. 28, pp. 115-123.
9. Das, S., Forer, L., Schönherr, S., Sidore, C., Locke, A.E., Kwong, A., Vrieze, S.I., Chew, E.Y., Levy, S. and McGue, M. (2016) Next-generation genotype imputation service and methods. *Nature genetics*, **48**, 1284.
10. O'Connell, J., Gurdasani, D., Delaneau, O., Pirastu, N., Ulivi, S., Cocca, M., Traglia, M., Huang, J., Huffman, J.E. and Rudan, I. (2014) A general approach for haplotype phasing across the full spectrum of relatedness. *PLoS genetics*, **10**, e1004234.
11. Kuleshov, M.V., Jones, M.R., Rouillard, A.D., Fernandez, N.F., Duan, Q., Wang, Z., Koplev, S., Jenkins, S.L., Jagodnik, K.M., Lachmann, A. *et al.* (2016) Enrichr: a comprehensive gene set enrichment analysis web server 2016 update. *Nucleic Acids Research*, **44**, W90-W97.
