# Supplementary material for: CSVS, a crowdsourcing database of the Spanish population genetic variability
Source: Nucleic Acids Res. 2020 Sep 29;49(D1):D1130–7. doi: 10.1093/nar/gkaa794 (PMC7778906; doi:10.1093/nar/gkaa794)
Supplement: gkaa794_Supplemental_Files [file gkaa794_supplemental_files.zip › Author's response.docx]

Dear Dr. Ridgen,

We are very happy for your recent email (4/09/2020) accepting the manuscript without any further review, and suggesting to promote some supplementary information to the main text. Actually, this is the first time that we have had this amazing experience. I must say that we have been working in this database for years and the manuscript has undergone a long maturation period. In any case we appreciate very much the referee’s nice comments and your kindness allowing more space for the parts moved from the supplementary material.

We have uploaded the new version, marking in red the parts that have been added from the supplementary material. We have renumbered the figures and reconstructed the supplementary material as well.

Please do not hesitate to contact us on any matter that may arise.

With my best wishes

Joaquin Dopazo, PhD
